# Identification of the PA1113 Gene Product as an ABC Transporter Involved in the Uptake of Carbenicillin in *Pseudomonas aeruginosa* PAO1

**DOI:** 10.3390/antibiotics9090596

**Published:** 2020-09-11

**Authors:** Christian Hulen, Pierre-jean Racine, Sylvie Chevalier, Marc Feuilloley, Nour-Eddine LOMRI

**Affiliations:** 1 Laboratory of Microbiology Signals and Microenvironment, EA 4312, Rouen Normandy University, 27000 Evreux, France; hulen.marie@orange.fr (C.H.); pierre-jean.racine@univ-rouen.fr (P.-j.R.); sylvie.chevalier@univ-rouen.fr (S.C.); marc.feuilloley@univ-rouen.fr (M.F.); 2 Département of Biology, Cergy-Pontoise University, 95000 Cergy-Pontoise, France

**Keywords:** ABC transporter, *Pseudomonas aeruginosa*, verapamil, carbenicillin

## Abstract

The resistance of *Pseudomonas aeruginosa* to antibiotics is multi factorial and complex. Whereas efflux pumps such as MexAB-OprM have been thought to predominate, here we show that a novel ATP Binding Cassette (ABC) transporter that mediates influx of carbenicillin from the periplasm to the cytoplasm and away from its cell wall target plays an important role in the resistance of *P. aeruginosa* to this antibiotic. Treatment of *P. aeruginosa* with verapamil, an inhibitor of ABC transporters in eukaryotic cells, increases its sensitivity to carbenicillin. Using amino acid sequence homology with known verapamil protein targets as a probe, we determined that the PA1113 gene product, an ABC transporter, mediates carbenicillin uptake into the bacterial cytoplasm. Docking and pharmacological analyses showed that verapamil and carbenicillin compete for the same site on the PA1113 gene protein, explaining the inhibitory effect of verapamil on carbenicillin uptake, and furthermore suggest that the PA1113 ABC transporter accounts for about 30% of *P. aeruginosa* carbenicillin resistance. Our findings demonstrate that the PA1113 gene product helps mediate carbenicillin resistance by transporting it away from its cell wall target and represents a promising new therapeutic target.

## 1. Introduction

Increasing bacterial resistance to antibiotics represents a worldwide health threat [1]. Resistance is believed to involve four separate mechanisms acting independently or in concert: (i) mutations in the genes encoding for antibiotic targets render them insensitive to drug action; (ii) changes in plasma membrane permeases and/or antibiotic trapping in the cell wall or surrounding biofilm which impede bacterial entry [2]; (iii) new genetic information acquired via plasmids or transposons encodes for inactivating enzymes such as β-lactamases [3,4]; and (iv) efflux pumps that expel antibiotics before they reach their targets.

Efflux pumps are generally homopolymers that belong to five protein super families [5]. The most important of these are comprised of ATP-binding cassette (ABC) transporters that mediate the influx of metabolites as well as efflux of drugs and toxins [6,7]. SMR (small multidrug resistance), a subfamily of DMT (drug metabolite transporters superfamily) and MATE (multi-antimicrobial extrusion), a subfamily of MOP (oligosaccharidyl multidrug-lipid polysaccharides flippases) can be found in some species. The two families of efflux pumps most represented in bacteria, the MFS (major facilitator) and RND (resistance nodulation division) super-families, are driven by transmembrane ion gradients. MFS is present in both Gram-positive and Gram-negative bacteria whereas the RND system, characterized by its broad spectrum of transported substrates, is present only in Gram-negative bacteria [8,9,10].

*Pseudomonas aeruginosa*, an adaptable bacterial saprophyte found in soil, water, sewage, and plants that is capable of causing serious respiratory, urinary, soft tissue, bone, and joint infections [11,12,13], has been identified by the World Health Organization (WHO) as one of the three most important microorganisms with respect to antibiotic resistance [14]. Antibiotic resistance by *P. aeruginosa* is believed mainly attributable to low outer membrane permeability and the expression of efflux pumps. The major outer membrane porin, OprF, the most important porin responsible for antibiotic exclusion by *P. aeruginosa* [15], exists largely in the closed configuration [16,17].

In addition to membrane impermeability, the resistance of *P. aeruginosa* is often associated with the presence of efflux pumps expressed constitutively or in response to antibiotic selection pressure [18,19]. Six RND systems have been identified in *P. aeruginosa*. The major system MexAB-OprM is frequently expressed in wild type strains [20]. The other systems, MexCD-OprJ, MexEF-OprN, MexJK-OprM, and MexXY-OprM, are often silent in wild-type strains and variably expressed in clinical isolates [21,22]. Recently, MexGHI-OpmD involved in the efflux of xenobiotics, heterocycle dye acriflavine, and norfloxacin [23] has been identified.

Proteins of the RND family have been the most studied in *P. aeruginosa* because they represent a major bacterial resistance mechanism; however, there are also others. An in silico analysis of the 5570 ORFs in the *P. aeruginosa* (PAO1) genome has shown that 44 ORFs could encode proteins involved in susceptibility and antibiotic resistance with a quasi-zero sequence evolution rate in amino acids [24] and 34 encode efflux systems that could mediate drug resistance, some of which are ABC transporters. However, relatively little information is available regarding the role of ABC ATP-binding cassettes in the transport of drugs for both their entry and exit.

An analysis of the nucleotide sequences of the PAO1 genome [25] suggests that more than a hundred ORFs encode ABC transporters. Some have been identified as necessary for the efflux of secreted proteins (AprD, hasD), siderophores (pyocheline), or lipid A (MsbA) and others for the influx of metabolites (amino acids, peptides, iron) [PAO1 genome data base, Pseudomonas.com]. These transporters can be exploited by antibiotics to circumvent natural resistance *P. aeruginosa* mechanisms [26]. For example, Pletzer et al. [27] have shown that microcin C, an antibiotic belonging to the peptide-nucleotide family, coopts the operon NppA1A2BCD, encoding the third PepT peptide transport system, to gain entry to *P. aeruginosa*. ABC carriers may also mediate efflux of antibiotics, as exemplified by the PA2812 gene product, UniprodKB Q91031, which mediates the efflux of ciprofloxacin [28].

Bacterial ABC transporters exhibit homology with those of eukaryotes, particularly in the highly conserved nucleotide binding domain (NBD) [6]. It has been shown that bacterial transporters can functionally complement that of eukaryotic cells, as is the case with the ABC transporter LmrA from Lactococcus lactis, which can functionally replace Multidrug-Resistance P-glycoprotein gene 1 (Mdr1) in human cells [29]. This strong homology also enables inhibitors of ABC transporters in eukaryotic cells such as the calcium channel blocker verapamil to work similarly in bacteria. Verapamil, for example, inhibits the efflux of acriflavin from Enterococcus faecalis EfrAB [30] as well as the export of rhodamine 6G from Bacillus subtilis [31].

Carbenicillin enters the periplasm through the water-filled porin channels in the outer-membrane [32] and is exported from the cytoplams by the export pump, MexAB-OprM. In the present study, we utilized verapamil [33] to further probe *P. aeruginosa* resistance mechanisms and our findings implicate a previously unrecognized import protein as playing an important role.

## 2. Results

### 2.1. Evaluation of the Response of P. aeruginosa PAO1 to Antibiotic Exposure in the Presence of Verapamil

Verapamil is used in clinical therapy as an inhibitor of calcium channels in the treatment of cardiovascular diseases such as angina pectoris, hypertension, and cardiac arrhythmias [34]. It is also used in anti-cancer therapies as an inhibitor of the main efflux pump that removes drugs from cancer cells, the membrane P-glycoprotein (P-gp) responsible for multidrug resistance (MDR phenotype) [33,35]. P-gp belongs to the large family of ABC transporters that hydrolyze ATP to catalyze the translocation of substrates across the cell membrane [36].

Our interest focused on the search of ABC transporters of *P. aeruginosa* involved in the resistance to antibiotics and in particular to carbenicillin. For this, we investigated whether verapamil had an effect on the susceptibility or resistance of the bacterium to this antibiotic.

**Figure 1:**
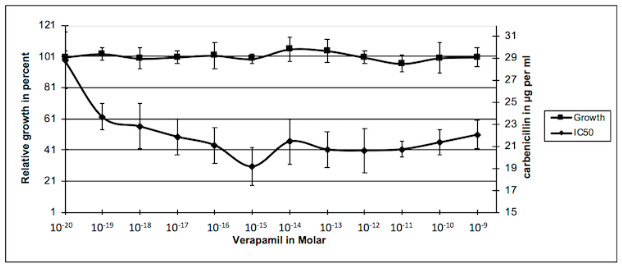
Effects of verapamil on *P. aeruginosa* growth and its sensitivity to carbenicillin.

Our preliminary experiments showed that verapamil had an effect on the sensitivity to the antibiotic at concentrations below 10^−9^ M. Figure 1 shows that verapamil did not inhibit the growth of PAO1 at concentrations ranging from 10^−20^ to 10^−9^ M. However, an effect on the growth of bacteria appeared for verapamil concentrations greater than 10^−6^ M. It was found that due to the very low solubility of verapamil at high concentrations in aqueous medium, it is difficult to differentiate the effect of verapamil from that of the solvent in which it is dissolved. For our study, we worked with verapamil concentrations ranging from 10^−20^ M (equivalent to the absence of molecules in the bacterial growth measurement wells) to 10^−9^ M (approximately 6 × 10^10^ verapamil molecules per well).

Growth: 100 µL Luria-Bertani (LB) liquid medium containing 1 × 10^5^ bacteria and increasing concentrations of verapamil were added in each well of 96 well plates. After 24 h incubation at 37 °C in a wet chamber, growth was estimated by measuring the absorbance at 595 nm and was compared to the control without verapamil. The results were plotted against concentration in alkaloid in the corresponding well. Experiments were repeated at least four times. IC_50_: *P. aeruginosa* PAO1 was inoculated in 96 well plates with increasing concentrations in verapamil (lines) and carbenicillin (columns) in 100 µL LB-medium. After 24 h incubation at 37 °C, bacteria growth was estimated in each well and reported on growth without antibiotic for each verapamil concentration. Relative growth was plotted against concentration in carbenicillin and the IC_50_ was calculated for each concentration in verapamil from the equation of the curve. Then, the obtained values were plotted against the concentration of verapamil. The presented results are the mean values of at least four independent experiments.

Figure 1 also shows the effect of verapamil on the sensitivity of *P. aeruginosa* to carbenicillin. The sensitivity of PAO1 (reduction of the IC_50_) to carbenicillin increased as a function of the augmentation in verapamil concentration. The IC_50_ decreased from 10^−19^ M to 10^−15^ M of verapamil concentration to reach a plateau. Beyond this concentration, sensitivity to the antibiotic did not vary significantly and formed a plateau. The appearance of this plateau is characteristic of a saturable system. Moreover, this system is not a major system in the development of the resistance of PAO1 to carbenicillin because the increase in sensitivity does not exceed 28% and in our experimental conditions, the addition of 60,000 molecules of verapamil in the wells was sufficient to obtain a maximum inhibitory effect of this system.

Verapamil is known as an inhibitor of efflux pumps of the ABC transporter family in eukaryotes. We can therefore hypothesize that the increase in sensitivity to carbenicillin observed here is related to the inhibition of an ABC system involved in the transport of this antibiotic. This system could act in conjunction with the MexAB-OprM system of PAO1 described in the literature as the major or even the single system of β-lactams efflux except for imipenem [37].

### 2.2. Search for the Target Protein of Verapamil

Our approach to the problem has been to carry out an in silico analysis based on the homology between amino acid sequences representing protein domains highly conserved and crucial for the function. Prokaryotic ABC transporters are polypeptides composed of 600 to 700 amino acids arranged in homopolymers to form influx or efflux pumps in the cytoplasmic membrane. Each monomer has a transmembrane domain and a cytoplasmic domain, while eukaryotic ABC transporters have a single polypeptide chain of more than 1250 amino acids, defining two domains. Each of them has a transmembrane and a cytoplasmic part and the two transmembrane parts joining to form the channel [38].

We recovered the amino acid sequences forming each domain of the rat protein Mdr1b, the eukaryote target for verapamil, and we performed a Basic Local Alignment Search Tool-Protein (BLAST-P) against proteins of *P. aeruginosa* PAO1 (Pseudomonas.com). With domain 1 of Mdr1b, the highest score was obtained with the product of the PA1113 gene, which encodes a putative ABC transporter (code access UniprotKB Q9I4M). With domain 2 of Mdr1b, the best score highlighted the product of the PA4997 gene that encodes the protein MsbA, an ABC transporter involved in the flip of lipid A-core moiety to the periplasmic side of the inner membrane [39], and the second score for the product of the PA1113 gene. The same analysis with the amino acids sequence of the human P-gp protein, equivalent to the rat Mdr1b, revealed the PA4143 gene product that encodes a putative ABC transporter for toxins and bacteriocins (code access UniprotKB Q9HWN8). Finally, moving toward prokaryotic proteins, we performed a BLAST-P against PAO1 proteins using LmrA from *Lctococcus lactis* [40] and BmrA from *Bacillus subtilis* [41] amino acid sequences. The highest score was obtained for the product of the PA3228 gene that encodes a putative ABC transporter (code access UniprotKB Q9HZ12). Table 1 shows the percentage of identity between these different proteins. On average, about 30% of identity is noted between these ABC transporters, whether they are of bacterial or eukaryotic origin, which shows a high level of conservation within this family.

As observed with amino acid sequence alignments on Serial cloner, the strongest identity was associated with the cytoplasmic Nucleotide Binding Domain (NBD) for ATP binding where the signature of specific sequences of the ABC carrier family is found. On the other hand, when the amino acid sequence of the major system of *P. aeruginos*is was compared to that of MexB, the percentage of identity was very weak, as shown in Table 1. MexB, which is used for the efflux of β-lactams, belongs to the family of RNDs that function differently from ABC transporters using the difference of potential between the two faces of the cytoplasmic membrane for substrate transfer and not ATP hydrolysis. We therefore focused on the products of the three genes PA1113, PA3228, and PA4143 as potential targets for verapamil. The PA4997 gene product, protein MsbA, was not retained as a candidate because the PAO1 mutant collection does not contain such a mutant. However, we obtained several mutants of the three other genes from this collection [42,43].

**Figure 2:**
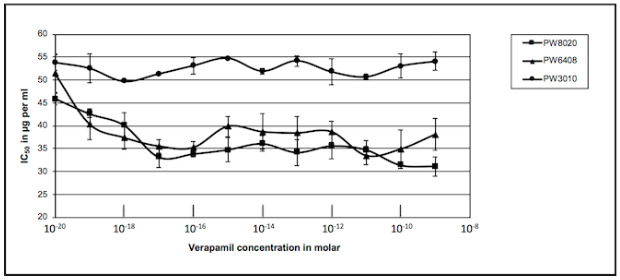
Effects of verapamil on sensitivity of several PAO1 mutants to carbenicillin. *P. aeruginosa* PAO1 mutants Pseudomonas Washington (PW) mutant PW3010, PW6408, and PW8020 were inoculated in 96 well plates with increasing concentrations in verapamil and carbenicillin. Growths and IC_50_ values were measured as described in the legend of Figure 1. The IC_50_ values were plotted against concentration in verapamil: PW8020 ■, PW6408 ▲, PW3010 ●. Results presented were the mean values of at least four independent experiments.

Mutants of candidate genes were tested for their sensitivity to carbenicillin in the presence of verapamil and their growth compared to that of the isogenic wild strain PAO1 treated under the same conditions. The values obtained for the IC_50_ are shown in Figure 2.

We can note that two of the three mutants exhibited the same decrease in IC_50_ as the isogenic PAO1 strain, while the third one showed no variation. To facilitate the analysis of these results, we calculated the mean value of the decrease of the IC_50_ on the plateau and reported these values in Table 2. A first analysis shows that the insertion of the transposon bearing the tetracycline resistance gene in the candidate genes substantially increased resistance to carbenicillin in the absence of verapamil (left column). The results shown in the right column of this table also show that the KO-mutation in the genes PA3228 and PA4143 did not change the response of the mutants compared to the wild strain. A decrease in the IC_50_ of 29% and 45%, respectively, was noted for a 28% decrease for the PAO1 strain. However, inactivation of the PA1113 gene by insertion of the transposon did not alter the mutant sensitivity to carbenicillin in the presence of verapamil. It appeared that the target of the alkaloid was absent in this mutant and its effect was eliminated.

**Figure 3:**
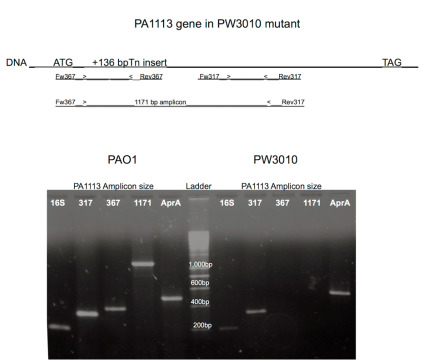
Expression of the PA1113 gene in PAO1 and its mutant PW3010. (**Top**) Schematic representation of the PA1113 gene. +136 bp after the ATG, shows the insertion position of the transposon (ISlacZ/hah). Below are the amplicons that were synthesized by RT-PCR to check the expression of the gene. (**Bottom**) The results generated by PCR from cDNAs obtained after reverse transcription on the total RNAs of PAO1 (on the left) and its mutant (on the right). RT-PCR was performed on total RNAs as described in the Materials and Methods section.

We tested the expression of the PA1113 gene in the wild-type strain PAO1 and its mutant PW3010 by Reverse Transcriptase—Polymerase Chain Reaction (RT-PCR.) Figure 3 (top) shows a schematic representation of the PA1113 gene with the position of insertion of the transposon at 136 base pairs downstream the ATG as well as the position of the primers used to check the expression of the gene and the size of the expected amplicons. As controls, expression of the 16S rRNA (rDNA) and the alkaline protease (AprA) genes was investigated. Figure 3 (lower part, left panel) shows the Polymerase Chain Reaction (PCR) result of PA1113 gene expression in PAO1 with the presence of the 367 bp amplicon on the 5 ‘side, the 317 bp amplicon in the middle of the gene, and the 1171 bp amplicon representing 2/3 of the gene. In the case of the PW3010 mutant, only the 317 bp amplicon was found. The insertion of the transposon in position +136 interrupts the gene in its 5′ side, causes the relocalization of the sets of primers, and no longer allows the synthesis of the 367 and 1171 bp amplicons as well as the synthesis of the expected mRNAs. However, the transcription of the PA1113 gene located downstream of the transposon, and therefore the appearance of the 317 bp amplicon after RT-PCR, could be explained by the presence of promoters on the 3 ‘side of the transposon.

**Figure 4:**
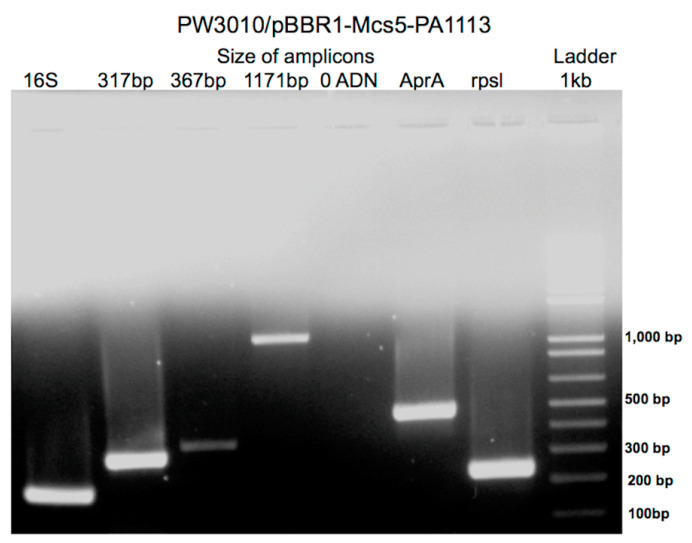
Expression of the PA1113 gene in the mutant PW3010 transformed with the plasmid pBBR1-Mcs5 containing the wild-type gene PA1113. RT-PCR was performed on total RNAs as described in the Materials and Methods section.

We then sought to carry out the functional complementation of this mutant. For this, we synthesized the wild-type gene PA1113 by PCR and cloned it in plasmid pBBR1-MCS5. The transformation of the mutant PW3010 with the recombinant plasmid containing the wild PA1113 gene makes it possible to recover normal expression of the gene, as shown by the results of the RT-PCR presented in Figure 4.

The three amplicons synthesized by RT-PCR from mRNAs extracted from the transformed mutant are identical to those obtained with the PAO1 strain having a wild type phenotype, except that the 317 bp amplicon is in greater quantity than the other two. This could be because the part of the gene downstream of the transposon insertion site is transcribed in the mutant to which are added the transcripts of the wild-type gene provided by the recombinant plasmid.

**Figure 5:**
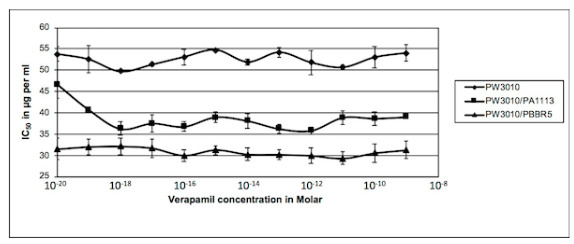
Effects of verapamil on sensitivity to carbenicillin of mutant PW3010 and PW3010 transformed either with pBBR5-PA1113 or the empty vector. PAO1 PW3010 mutant (♦), PW3010 transformed with plasmid pBBR5 containing the wild PA1113 gene (■), and PW3010 transformed with the empty vector pBBR5 (▲) were inoculated in 96-well plates with increasing concentrations of verapamil and carbenicillin. Growths and IC_50_ values were measured as described in the legend of Figure 1. The IC_50_ values were plotted against concentration in verapamil. Results presented were the mean values of at least four independent experiments.

As shown in Figure 5, the transformation of the mutant PW3010 with the recombinant plasmid carrying the wild PA1113 gene restored the sensitivity to carbenicillin in the presence of verapamil with a decrease in the IC_50_ of 22% (Table 2, line 5).

As the control experiment, the transformation of the mutant with the empty vector did not allow for the modification of the mutant sensitivity to carbenicillin in the presence of verapamil (Figure 5 and Table 2, line 6). The product of the PA1113 gene, a potential ABC transporter, seems to be the target of verapamil and is involved in the membrane transport of carbenicillin in *P. aeruginosa*.

### 2.3. Function of the PA1113 Gene Product

Inhibition of the ABC transporter, product of the PA1113 gene, by verapamil increases the sensitivity of *P. aeruginosa* to carbenicillin. However, is this ABC transporter involved in the uptake of carbenicillin in the bacterial cytoplasm or in its efflux? The target of the antibiotic is a periplasmic penicillin binding protein involved in the final step of condensation of the peptides interconnecting peptidoglycan chains to provide rigidity of the assembly. If we hypothesize that this ABC transporter is involved in the influx of carbenicillin, blocking the function by binding the verapamil should lead to an accumulation of the antibiotic in the periplasm. An increase, even transitory, of the intra-periplasmic concentration of carbenicillin should facilitate access of the antibiotic to its target and make the bacteria more sensitive.

To test this hypothesis, we performed the kinetics of carbenicillin input into the periplasm of PAO1 in the presence or absence of verapamil in a short time, recovered the periplasm by the action of Ethylene Diamine Tetraacetic Acid (EDTA) and lysozymes, and then measured the amount of carbenicillin. For this assay, we carried out a standard growth inhibition curve of the sensitive strain *Bacillus subtilis* 168 to carbenicillin through tests in wells on LB agar plates.

**Figure 6:**
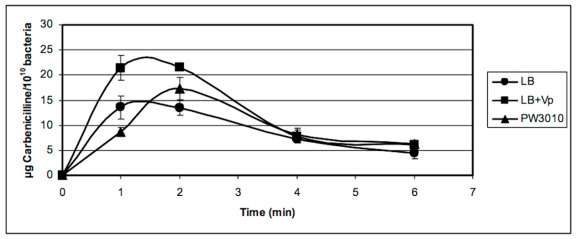
Kinetics of accumulation of carbenicillin in the periplasm of *P. aeruginosa* PAO1 and its mutant in the PA1113 gene (PW3010) in the presence (LB + Vp) or absence (LB and PW3010) of verapamil. *P. aeruginosa* PAO1 suspended in LB medium or LB + 1 × 10^−7^ M verapamil at a concentration of 1 × 10^10^ bacteria per mL was incubated for 20 min at 37 °C before starting the kinetics. The kinetics and recovery of periplasmic contents were carried out as described in the Materials and Methods section. The amount of carbenicillin in periplasmic extracts was determined from a standard range established with the high sensitive bacterium *Bacillus subtilis* 168 and plotted against time. The results presented in the figure are the mean values of at least three independent experiments.

Figure 6 shows an accumulation of carbenicillin in the periplasm for the first 2 min before the antibiotic enters the cytoplasm or gets out through porins. We also noted that the accumulation of the antibiotic in the periplasm was much higher when the bacteria were treated with verapamil rather than in its absence. The mutation in the PA1113 gene also led to the accumulation of more carbenicillin in the first 2 min in the periplasm than the isogenic strain PAO1.

TheABC transporter,expression productof thePA1113gene, should allow for the entry of carbenicillin in the cytoplasm. Its temporary blockage by verapamil would increase the antibiotic concentration in the periplasm, thereby facilitating its interaction with the penicillin binding protein, its target protein. As shown in Figure 6, the accumulation of carbenicillin in the periplasm ceases after 4 to 5 min. This can be explained by the establishment of equilibrium between the entry of the antibiotic in the cytoplasm by several carriers such as the PA1113 gene product and the release of the antibiotic by one or more porines by facilitated diffusion.

**Figure 7:**
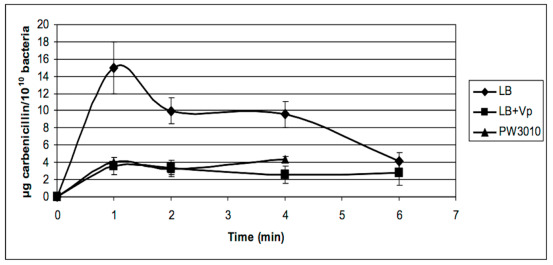
Kinetics of accumulation of carbenicillin in the cytoplasm of *P. aeruginosa* PAO1 in the presence (LB + Vp) or absence (LB) of verapamil and in the mutant in PA1113 gene (PW3010). *P. aeruginosa* PAO1 and its mutant PW3010 suspended in LB medium or LB + 1 × 10^−7^ M verapamil at a concentration of 1 × 10^10^ bacteria per mL were incubated for 20 min at 37 °C before starting the kinetics. After recovery of the periplasmic contents, PAO1 protoplasts were lysed by sonication and the cytoplasmic contents were recovered after centrifugation. The amount of carbenicillin in cytoplasmic extracts was determined from a standard range established with the high sensitive bacterium *Bacillus subtilis* 168 and plotted against time. The results presented in the figure are the mean values of at least three independent experiments.

Figure 7 shows the results of the kinetics of accumulation of carbenicillin in the cytoplasm of *P. aeruginosa* PAO1 in the absence or presence of verapamil. Carbenicillin penetrated four times less in the cytoplasm of bacteria treated with verapamil during the first minute than in untreated bacteria before equilibrium was reached after 6 min.

The results obtained with the mutant PW3010 of the PA1113 gene (Figure 7) was identical to that obtained with PAO1 in the presence of verapamil. These observations confirm the function of the gene product PA1113 as an ABC transporter used for drug influx, allowing the entry of carbenicillin into the cytoplasm of the bacterium.

**Figure 8:**
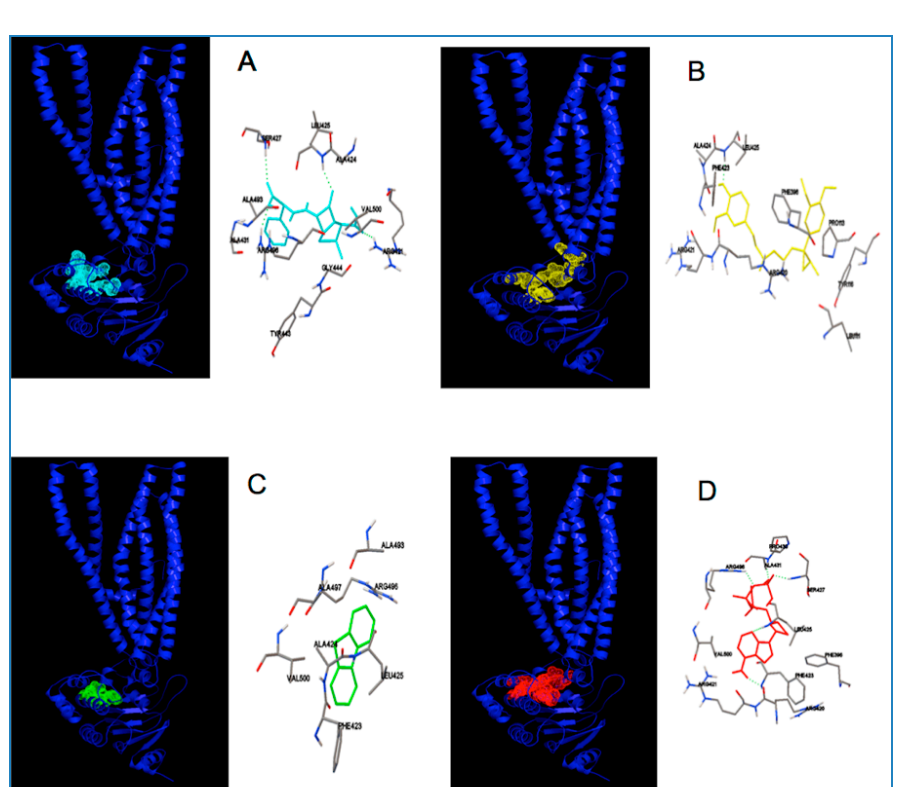
Modeled interactions of the PA1113 ABC transporter with carbenicillin (light blue), verapamil (yellow), dibenzothiophene (green), and ATP (red). (**A**–**D**) show the general views of the binding of the PA1113 gene product to carbenicillin, verapamil, dibenzothiophene, and ATP, respectively. Detailed views of the binding site showing the principal amino acids potentially involved in the binding of carbenicillin, verapamil, dibenzothiophene, and ATP; ARG421, PHE423, ALA424 and LEU425 are implicated in binding with the different substrates.

Molecular modelization of the PA1113 gene product (UniProtKB-Q914M2) revealed that carbenicillin and verapamil should interact on a common site (Figure 8). Detailed analysis of the binding pocket for the two molecules showed that at least three amino acids of PA1113; ARG421, ALA424, and LEU425 appear essential for the binding of carbenicillin and verapamil.

## 3. Discussion

Like many bacteria responsible for nosocomial infections, *Pseudomonas aeruginosa* is naturally resistant to many antibiotics, particularly through the presence of nine efflux systems assigned to the RND family including the six Mex-systems that confer significant resistance to antibiotics [44]. However, several other elements can contribute to the establishment of a multidrug resistance phenotype including ABC transporters such as that recently identified by Pletzer et al. (2015) [27] for uptake of peptidyl nucleoside antibiotics.

To investigate the involvement of ABC transporters in the resistance of *P. aeruginosa* to β-lactams, we used an alkaloid identified as an inhibitor of ABC transporters in animal cells, verapamil. The treatment of *P. aeruginosa* with this alkaloid makes the bacteria more susceptible to carbenicilin. Due to the high specificity of verapamil, the bacterial ABC transporter may be involved in the resistance of *P. aeruginosa* to this antibiotic. From the amino acid sequences of eukaryotic proteins identified as the target for verapamil and two ABC transporters from Gram-positive bacteria, LmrA and BmrA, we performed a BLAST-P against the total proteins of *P. aeruginosa* PAO1. By performing the BLAST-P from proteins and target sequences of our alkaloid on proteins of *P. aeruginosa* PAO1, we identified four potential candidates among hundreds of ABC transporters that belong to this bacterium.

By focusing our study on the sensitivity to carbenicillin in the presence of verapamil, we found that the mutant of the PA1113 gene no longer presented any variation in sensitivity to the antibiotic unlike the isogenic wild strain. The introduction of a wild-type gene in this mutant brought a functional complementation with recovery of the increased sensitivity to carbenicillin in the presence of verapamil.

**Figure 9:**
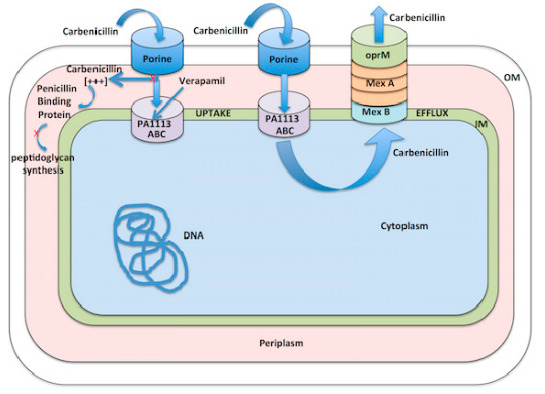
Proposed mechanism for carbenicillin influx.

The kinetic study of the entrance of carbenicillin in the bacterium PAO1 with or without verapamil showed that the product of the PA1113 gene is an ABC transporter involved in the entry of the antibiotic into the cytoplasm. Its expulsion by the MexAB-OprM system has been described in the literature [20,37]. Indeed, in the presence of verapamil, there is a transient accumulation of carbenicillin in the periplasm that promotes the interaction of the antibiotic with its target, a penicillin binding protein. This accumulation of even a few minutes is sufficient to cause inhibition of peptidoglycan synthesis and induces the death of bacteria, expressing a significant decrease in viable bacteria in the early hours of growth (Figure 9).

The PA1113 gene product should be an influx protein. This function has been raised by Noda et al. (2003) [45], who observed a loss of degradation of dibenzothiophene in medium n-tetradecane by a mutant of *P. aeruginosa* in the PA1113 gene. Since dibenzothiophene could not penetrate any more in the bacterial cytoplasm, desulfurization could not take place. Like carbenicillin, dibenzothiophene is characterized by a sulfur atom bonded to two carbon atoms involved in a cycle for which all or part can enroll in a plan. As shown in Figure 8, dibenziothiphene could bind on the same site as carbenicillin, involving recognition of two common amino acids, namely ALA424 and LEU425. In addition, it is interesting to note that the key amino acid in the binding of these molecules, LEU425, also appears as one of the principal amino acids required for interaction of ATP with the ABC transporter (Figure 8). The formation of specific interactions with the common amino acids involved in the binding site of the ligands to the ABC transporter could explain their uptake as well as their inhibition with verapamil.

Carbenicillin enters the periplasm through the water-filled porin channels in the outer-membrane [32], enters the cytoplasm via PA1113 gene product (our findings), and is exported from the cytoplams by the efflux pump, MexAB-OprM. In the present studies, we utilized verapamil to further probe *P. aeruginosa* resistance mechanisms and our findings implicate a previously unrecognized import protein as playing an important role. Figure 9 shows our proposed mechanism for carbenicillin influx and its inhibition by verapamil.

We have shown in this study that the use of an ABC transporter inhibitor may increase the efficacy of carbenicillin, but not enough to totally eliminate the bacteria. This ABC transporter is implicated in the uptake of the antibiotic. Research and development of new ABC transporter inhibitors as well as RND inhibitors, obtained either by synthesis or extraction from nature, are promising approaches for the implementation of a combination therapy involving both pump inhibitors and antibiotics, as shown by others [46,47].

## 4. Materials and Methods

### 4.1. Bacterial Strains and Growth Conditions

The genus and strains used in this study are listed in Table 3.

Strains were stored at −80 °C in LB containing 20% glycerol. Bacteria were routinely grown in Luria broth (LB) medium and on LB agar plates or LB agar plates plus tryptophan for *B. subtilis* for 24 h at 37 °C.

### 4.2. Sensitivity of P. aeruginosa to Carbenicillin in the Presence of Verapamil

The sensitivity of *P. aeruginosa* to carbenicillin and verapamil (Sigma Aldrich, France) was studied using 96-wells plates containing 100 µL per well with increasing concentrations of the antibiotic and alkaloid. The size of the inoculums (1 × 10^5^ bacteria per well) was calibrated to reach the end of the exponential growth after 24 h. The plates were incubated 24 h at 37 °C in a wet chamber. The absorbance at 595 nm in each well was measured at t_0_ and at t_24_ in a plate reader µQuant (Bio-Tek instruments, Inc., Winuski, VT, USA). After incubation, bacterial growth was estimated from the difference of absorbance at 595 nm. For each concentration of drug and antibiotic, the relative growths were calculated as the ratios of the absorbance value in the wells to that of the control (first row), and the results were plotted against the concentration in carbenicillin. The minimal concentration of antibiotic that inhibited 50% of growth (IC_50_) was calculated from the equation of the curve for each concentration in verapamil and plotted against the related drug concentrations.

Sensitivity to carbenicillin in the presence of increasing concentrations in verapamil was tested on PAO1, mutants PW3010, PW6408, PW8020, and the PW3010 mutant transformed with the plasmid recombinant pBBR1-PA1113 or with the empty vector (Table 2).

### 4.3. General DNA Procedures

Restriction enzymes and T4 DNA ligase were purchased from New England Biolabs (Ipswich, MA) and used according to the manufacturer’s instructions. Plasmid and primers used for PCR experiments are listed in Table 4.

Synthesis of the PA1113 gene by PCR was carried out with 1 µg of *P. aeruginosa* PAO1 chromosomal DNA, 20 pmol of each primer, and Taq DNA polymerase (Roche Molecular Biochemicals). Primers used for the synthesis of the amplicon of 2466 nt were Fw 5′-ATGATGGCCATATCCGGGGAC-3′ and Rev 5′-ACATCTAGATGAGCGTCAAGTACC-3′. This amplicon harbored an *Eco*RI site 200 nt upstream of the ATG and a *Xba*I site at the 3′ end 196 nucleotide downstream of the translation termination site. The *Eco*RI-*Xba*I-digested PCR product was inserted into *Eco*RI-*Xba*I-digested pBBR1-MCS5, yielding pBBR1-PA1113. *E. coli* DH5α was then transformed by the thermal shock procedure as previously described and the transformants were selected on LB agar plates containing 15 µg.mL^−1^ gentamycin. Recombinant plasmid pBBR1-PA1113 was purified from recombinant bacteria and tested for the presence of the right insert. Several positive clones on LB agar plates containing 15 µg.mL^−1^ gentamycin were tested by PCR on colonies using a set of primers (Table 4), allowing for the synthesis of a fragment 367 nt containing the mutagenic transposon insertion site. Positive clones have the expected amplicon as controlled after recombinant plasmid DNA analysis.

RT-PCR. Total RNA was extracted using the Extract All Kit (Eurobio) from an overnight culture of PAO1, mutant PW3010, and PW3010 positive clone containing the pBBR1-PA1113 plasmid. cDNA synthesis was performed using reverse transcriptase MFLV (Promega) in the presence of random primers according to the protocol recommended by the provider. PCRs using primers to cover the 5′ end and 2/3 of the gene (Table 4) were performed to check the expression of the PA1113 gene.

### 4.4. Accumulation of Carbenicillin in Bacteria

*P. aeruginosa* PAO1, PW3010 mutant, and PW3010 pBBR1-PA1113 were grown to confluence on LB-agar plates for 24 h at 37 °C. Bacteria were recovered by scraping with a sterile loop and resuspended in LB medium or LB medium containing verapamil 1 × 10^−7^ M, at a concentration of 1 × 10^10^ bacteria per mL. Two mL of bacteria were incubated 20 min at 37 °C before performing kinetics to allow the verapamil to interact with its target. Kinetics were established for the times of 1, 2, 4, and 6 min in Eppendorf tubes with 250 µg of carbenicillin per ml. At t + 6 min, the tubes were centrifuged for 3 min at 10,000 g and at 4 °C to stop the transport of the antibiotic. Supernatants were removed and the bacteria were resuspended in 500 µL of 50 mM Tris-HCl pH8 and 1 mM EDTA containing 50 µg of lysozyme. The bacteria were incubated at 37 °C and the formation of spheroplasts was monitored by using an optical microscope at a magnification 1000. After 30 min of incubation almost all bacteria changed shape. The bacteria were centrifuged for 15 min at 13,000 g. The supernatants for each time of kinetics were filtered through a sterile 0.2 µm filter to remove bacteria that had not sedimented. The pellets were resuspended in 500 µL of sterile water and bacteria were then broken by sonication in a Branson sonifier six times for 30 s in ice. The broken bacteria were centrifuged for 20 min at 15,000 g and the cytoplasmic contents were recovered.

**Figure 10:**
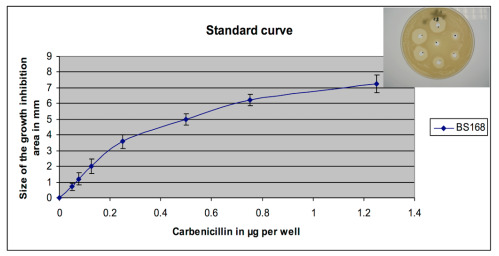
Range of sensitivity of *Bacillus subtilis* 168 to carbenicillin. About 1 × 10^6^ bacteria were spread on the LB-agar plate. After 1 h of incubation at 37 °C, holes were drilled in the agar with a sterile Pasteur pipette and 50 μL of a solution containing increasing quantities of carbenicillin were added to each well. After 24 h of incubation at 37 °C, the size of each halo of growth inhibition was measured and plotted as a function of the quantity of carbenicillin in the wells. Such a standard range was performed for each carbenicillin input measurement experiment in PAO1 or its mutant PW3010.

The presence of carbenicillin in the cytoplasmic and periplasmic extracts was detected by the inhibition of the growth of a test bacterium highly sensitive to carbenicillin, *Bacillus subtilis* 168. Approximately 1 × 10^6^ *B. subtilis* 168 were plated on LB-agar plates and incubated for 1 h at 37 °C. Wells of 5 mm in diameter were cut in the agar and 50 µL, 25 µL, 10 µL, and 5 µL of samples for each time of each kinetic were added to the wells and the volume completed to 50 µL with sterile water. For each experiment, a plate containing 7-wells was used to determine the sensitivity of *B. subtilis* 168 to carbenicillin and to establish a standard curve between 50 ng and 1.25 µg per well (Figure 10).

After overnight incubation at 37 °C, the diameter of growth inhibition areas around the wells was measured. With the control plate, a sensitivity calibration range to carbenicillin was established using the width of the halos of the inhibition (in mm) as a function of the amount of carbenicillin per well (in µg). For each experiment, the size of each halo was plotted on the reference range and the amount of carbenicillin in each well was determined. Each point of the kinetic values is expressed in µg carbenicillin per 1 × 10^10^ bacteria. All experiments were carried out at least three times.

### 4.5. Molecular Modelization

The FAST-All alignment tools amino acid sequences (FASTA) of the proteins were obtained from UniprotKB. Sequence alignment was performed with a Serial cloner and the percentage of identity was determined. The 3D structure of PA1113 protein (UniProtKB-Q914M2) was calculated by RaptorX [49] and visualized using Python Molecular Viewer V1.5.6. Potential interactions between ligands and the target protein were assayed using AutoDock 4 [50].

All calculations were performed using a DELL PowerEdge T420 computer equipped with four hard disks (4 To each leading to 12 To under RAID5).

## 5. Conclusions

In conclusion, we believe that it is virtually impossible to eradicate opportunistic pathogenic bacteria responsible for chronic infections or sepsis when they are resistant or multi-resistant to antibiotics. However, it should be possible to use a therapy directed against several bacterial targets to significantly reduce the microbial load so that the commensal flora and immune system can control the spread of infectious exogenous bacteria.

## Figures and Tables

**Table 1:** Percentage of identity between amino acid sequences of proteins performed using BLAST-P.

	PA1113	BMRA	Mdr1b	PA3228	PA4143	LMRA	MsbA	MexB
**PA1113**	100	33	30	30	24	31	35	18
**BMRA**	33	100	27	28	23	27	30	17
**Mdr1b**	30	27	100	27	22	29	30	16
**PA3228**	30	28	27	100	22	17	31	16
**PA4143**	24	23	22	22	100	21	22	14
**LMRA**	31	27	29	17	21	100	30	14
**MsbA**	35	30	30	31	22	30	100	15
**MexB**	18	17	16	16	14	14	15	100

**Table 2:** Effects of verapamil on the sensitivity of *P. aeruginosa* PAO1 and its mutants to carbenicillin.

Strain	Gene	None	IC_50_ (µg/mL) Mean Value
PAO1 Washington		29 ± 3.5	21 ± 2
PW8020	PA4143	42 ± 2	23 ± 1
PW6408	PA3238	55 ± 4	39 ± 4
PW3010	PA1113	54 ± 2	54 ± 1.5
PW 3010 Transformed	pBBR5-PA1113	48 ± 3	37.5 ± 1.5
PW 3010 Transformed	pBBR5	31.5 ± 2.5	30.5 ± 1.5

**Table 3:** *Pseudomonas aeruginosa* and other bacteria strains used in this study.

*Pseudomonas aeruginosa* Strains Used in This Study			
Strain	Characteristics/Modified Gene	Function	Reference or Source
MPAO1	Wild type serotype O		Washington UC collection
PW3010	PAO1 mutant in gene PA1113	probable ATP-binding/permease fusion ABC transporter	Washington UC collection
PW6408	PAO1 mutant in gene PA3228	probable ATP-binding/permease fusion ABC transporter	Washington UC collection
PW8020	PAO1 mutant in gene PA4143	probable toxin transporter	Washington UC collection
**Other Bacteria Used**			
*Escherichia coli* DH5α		F-Ф80dlacZΔM15(lacZYA-argF) U169disRrecA1endA1hsdR17(rk^−^mk^+^)gal^−^phoAsupE44λ^−^thi^−^gyrA96relA1	Invitrogen
*Bacillus subtilis* 168			

**Table 4:** Plasmid and primers used for PCR experiments.

Plasmid	pBBR1MCS-5 4768 bp Gentamycin [48]
DNA Synthesis	Primers Used in this Study
PA1113 gene synthesis	Fw 5′-ATGATGGCCATATCCGGGGAC-3′
	Rev 5′-ACATCTAGATGAGCGTCAAGTACC-3′
367bp amplicon	Fw 5′-TGCCAGCCATTCTCTCCTC-3′
	Rev 5′-CGGAGCTACCGTTGGTTTC-3′
317 bp amplicon	Fw 5′-CGTTCGTCTTCTACAGCCTG-3′
	Rev 5′-ATCGAACAGGGTCGACTTGC-3′
1171 bp amplicon	Fw 5′-TGCCAGCCATTCTCTCCTC-3′
	Rev 5′-ATCGAACAGGGTCGACTTGC-3′
16S RNA	Fw 5′-AAGCAACGCGAAGAACCTTA-3′
	Rev 5′-CACCGGCAGTCTCCTTAGAG-3′
AprA (alcaline protease)	Fw 5′-TACGGCTTCAACTCCAACAC-3′
	Rev 5′-TCGACGTATTGCAGCACCA-3′
rpsL (RNA pol sub-unit)	Fw 5′-GCAACTATCAACCAGCTGGTG-3′
	Rev 5′-GCTGTGCTCTTGCAGGTTGTG-3′
